# Paediatric Snakebite Toxicity up to Compartment Syndrome: A Ten-Year Retrospective Study in Iasi, Romania

**DOI:** 10.3390/toxins18030128

**Published:** 2026-03-02

**Authors:** Bogdan Caba, Sidonia Petronela Susanu, Ioana Cezara Caba, Tamara Roșu-Solange, Bogdan Huzum, Vasile Eduard Roșu, Ileana Katerina Ioniuc, Maria Adriana Mocanu, Iuliana Magdalena Starcea

**Affiliations:** 1 Grigore T. Popa University of Medicine and Pharmacy, 700115 Iasi, Romania; bogdan-caba@umfiasi.ro (B.C.); petronela-sidonia.susanu@umfasi.ro (S.P.S.); solange.rosu@umfiasi.ro (T.R.-S.); bogdan.huzum@umfiasi.ro (B.H.); eduard.rosu@umfiasi.ro (V.E.R.); ileana.ioniuc@umfiasi.ro (I.K.I.); maria-adriana.mocanu@umfiasi.ro (M.A.M.); iuliana.starcea@umfiasi.ro (I.M.S.); 2 Faculty of Medical Bioengineering, Grigore T. Popa University of Medicine and Pharmacy, 700115 Iasi, Romania; 3 Saint Mary Emergency Hospital for Children, 700309 Iasi, Romania; 4 Faculty of Medicine, Grigore T. Popa University of Medicine and Pharmacy, 700115 Iasi, Romania; 5 Faculty of Pharmacy, Grigore T. Popa University of Medicine and Pharmacy, 700115 Iasi, Romania; 6 “Sf. Spiridon” Emergency Clinical Hospital, 700111 Iasi, Romania

**Keywords:** snakebite, compartment syndrome, fasciotomy, toxicology, plastic surgery, local tissue toxicity, functional limb recovery, antivenom

## Abstract

Viper bites are medical emergencies that can develop into serious clinical complications and can endanger the life of the paediatric patient. This observational retrospective study analyses 24 cases of viper bites involving paediatric patients (<18 years) encountered over 10 years (2016–2025) in the emergency department of Saint Mary Emergency Hospital of Iasi, Romania, with a focus on those requiring specialised surgical monitoring. Sociodemographic factors, toxicity, and surgical management of snakebites were analysed. In 83.33% cases, viper bites were found on the lower limb. The retrospective study was completed through an in-depth analysis of two representative cases, with a particular focus on the evolution up to compartment syndrome. Of the 24 cases presented in the Emergency Department, one was a rare and severe case, which evolved into compartment syndrome and required fasciotomy to save the limb in the Plastic Surgical Department. Another one, with the bite localised at the upper limb, had perilesional oedema, without skin colour changes or secretions, preservation of joint contours, and normal nail colouration. Both were analysed and described in detail with all available data (images, investigations, etc.) to highlight pathophysiological and therapeutic aspects. Appropriate, multidisciplinary treatment considerably improves the functional prognosis of patients with viper bites; administration of antivenom and selective fasciotomy contribute to successful outcomes. The study emphasises that viper bites in children remain a public health problem in Romania and require prompt and multidisciplinary treatment.

## 1. Introduction

Viper bites are a major medical emergency in Central and Southeastern Europe, with variable public health impacts [1,2]. In Romania, the bites of three species—*Vipera ammodytes*, *Vipera berus* and *Vipera ursinii*—result in a spectrum of clinical symptoms ranging from minor local reactions to severe systemic complications [3]. Among these, *Vipera ammodytes* (the horned viper) is the species relevant to distribution in the northeastern part of the Carpatho-Pontic area and is most frequently encountered with serious manifestations, including coagulopathies, thrombocytopenia, and an increased need for antivenom [4,5].

In Central and SE Europe, approximately ≥2.55 bites/million inhabitants are estimated, and bites by *V. ammodytes* are reported to be more frequent and severe, with more symptoms such as pain, thrombocytopenia, and a greater need for antivenom [5]. *V. ammodytes* is associated with a higher risk of severe manifestations compared to other European species [2,4]. Romanian data mostly come from case reports, local clinical studies, and review articles (to the best of our knowledge, there is no published national registry focused solely on NE).

The severity of toxicity can be influenced by factors such as venom amount, bite depth, bite location, and patient age [6]. Compared to adult patients, paediatric patients, with smaller body size, lower total dilution volume, and physiological differences, are at higher risk of snakebite-related complications, more severe local lesions, systemic effects, and a higher rate of surgical intervention [6,7]. Children are more vulnerable than adults to snakebites, and their curious nature and lack of restraint and/or awareness put them at greater risk of poisoning [8]. Snakebites can cause major disabilities in children, with an impact on development and affecting the family from a social, economic and psychological point of view, something also mentioned in a study from the Brazilian Amazon, which describes the very impressive cases of three children after viper bites [9]. Consequently, recognition of local and systemic complications can lead to the successful treatment of these pathologies. Major complications described include compartment syndrome, coagulation disorders, acute renal failure and, rarely, neurotoxic manifestations or death [4,6,10]. Cardiovascular complications are not frequently described, but can become dangerous when not appropriately treated [11]. Cerebral and myocardial infarction can develop; myocarditis is very rare [11]. Symptoms such as oedema of the lips and tongue, angioedema and the need for oro-tracheal intubation, thrombocytopenia and anaemia have been described in a 33-year-old man by Maffè [11]. Although antivenom remains the main specific treatment, variations in its availability (sometimes, there is a temporary shortage of the antivenom) and use may influence patient prognosis [12].

In this context, a comprehensive evaluation of viper bite cases in Northeastern Romania is necessary to characterise the clinical patterns and risk factors associated with complications.

## 2. Results

### 2.1. General Characteristics of the Study Group (n = 24)

Patients and clinical features at emergency care unit presentation: A total of 24 cases of viper bites were identified over 10 years (2016–2025) at the emergency unit of Saint Mary Children’s Hospital, Iasi.

**Figure 1:**
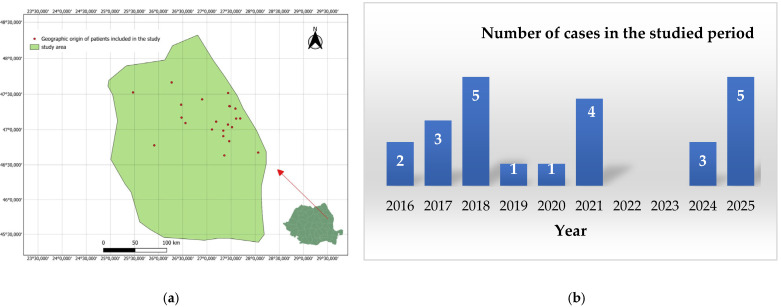
Characterization of the studied batch (**a**) geographic origin of snakebites included in the study (created with QGIS); (**b**) number of cases by year, between 2016 and 2025 (10 years), with 5 cases in 2018 and 2025.

Figure 1 shows the number of cases in the analysed period by year. Of these, 9 of 24 (37.5%) were aged 2–10 years, and 15 of 24 (62.5%) were aged 11–16 years. Five cases (20.83%) were found in urban areas and 19 (79.16%) in rural areas. Only 5 of 24 patients (20.83%) were female; the remaining 19 (79.16%) were male. Viper bites were found in 20 cases of 24 (83.33%) on the lower limb. In some cases, patients received antivenom serum (four patients) and/or tetanus vaccine (eighteen patients) (Table 1). The number of hospitalisation days until discharge ranged from 1 to 11 days, depending on the severity of intoxication.

The patients’ ages were, on average, 11 years (SD = 5), with values ranging from a minimum of 2 to a maximum of 16 years, and the duration of hospitalisation was, on average, 4 days (SD = 2), ranging from a minimum of 1 day to a maximum of 11 days.

In Figure 1a, a map of the geographic locations of patients included in the study showed the Northeastern part of Romania. Saint Mary Clinical Emergency Hospital in Iași, Romania, treats emergencies across all of these areas. Figure 1b shows that 2018 and 2025 had the highest number of snakebites.

**Figure 2:**
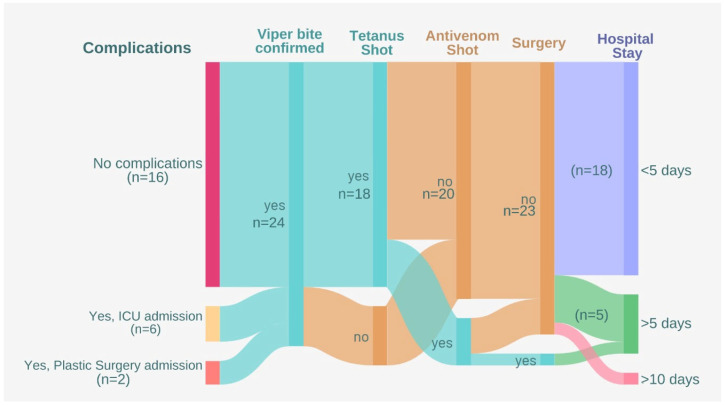
Alluvial diagram of the snakebite cases from 2015 to 2026, admitted in the Saint Mary Hospital (created with Flourish).

The hospitalisation flow was visualised and described in Figure 2 as an alluvial diagram.

The symptomatology described in the emergency unit following the viper bite in the paediatric patients included localised oedema, lymphangitis, excoriated wounds, punctate phlyctenular lesions, tachycardia, non-acidotic ketosis, allergoderma or post-bite anaphylactic reaction, acute dehydration syndrome through vomiting and diarrhoea, and compartment syndrome, which endangers the patient’s life.

Two cases of these snakebites required surgical management and were treated in the Plastic Surgery Department of the Saint Mary Children’s Emergency Hospital in Iasi, Romania; one of them was complicated by an extreme adverse reaction and compartment syndrome (treated with fasciotomies, repeated dressings, and secondary sutures).

**Figure 3:**
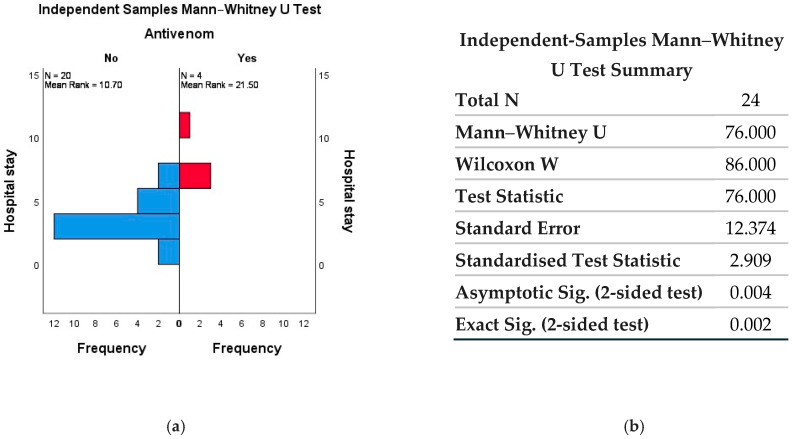
Mann–Whitney U Test for antivenom influence on length of hospital stay (**a**) distribution of length of hospital stay (days) by antivenom administration (No vs. Yes). The no antivenom group (N = 20) had a mean rank of 10.70, and the antivenom group (N = 4) had a mean rank of 21.50, suggesting a longer length of hospital stay in patients treated with antivenom; (**b**) summary of statistical analysis by Mann–Whitney U test for independent samples (total N = 24). The difference was statistically significant (U = 76, Z = 2.909, *p* = 0.004; exact *p* = 0.002), indicating a significant association between antivenom administration and length of hospital stay.

In Figure 3, statistically significant correlations were observed between antivenom administration and shorter hospital length of stay. Unfortunately, due to the small number of cases during the studied period, no other statistically significant correlations were identified, which limited our study.

### 2.2. Detailed Description of Cases Treated in the Plastic Surgery Department

#### 2.2.1. Patient 1—Compartment Syndrome in the Lower Limb with Fasciotomy

A 5-year-old patient, transferred from a rural mountain town in NE Romania, presents to the emergency centre with a viper bite in the left lower limb. On admission to the emergency unit, the clinical examination revealed a distressed facial expression, pale skin, tachycardia, and, on local examination, significant hard oedema of the left lower limb, functional impotence, extreme pain (especially after passive movements), and ecchymosis, with the affected limb being significantly larger and colder compared to the healthy one. Therefore, the natural evolution of the snakebite in this case was towards limb compartment syndrome in the absence of fasciotomy.

**Figure 4:**
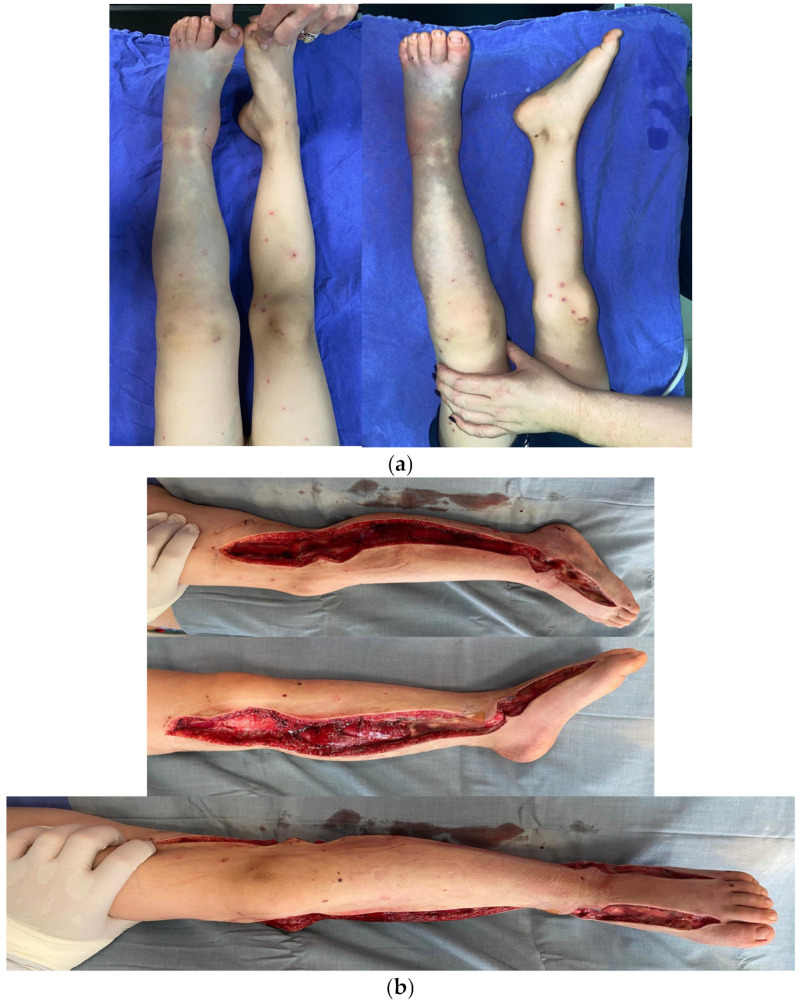
Aspects of the lower limb observed during hospitalisation and treatment: (**a**) on local examination, ischaemic damage; (**b**) during fasciotomy (a long anterolateral longitudinal incision, allowing complete decompression of the anterior and lateral compartments).

The patient was hospitalised urgently, received supportive care, and the compartment syndrome was treated by emergency fasciotomy. Due to the lack of a proper device, only clinical criteria were used when deciding the need for fasciotomy. The patient experiences severe pain during passive and active extension and flexion of the fingers and the lower leg. The patient has also lost cutaneous sensibility and has paraesthesia. This was performed on the anterior, lateral, and posterior muscle compartments of the calf and left leg (Figure 4). Haemostasis was achieved, and a negative wound dressing was applied. Coagulation disorders were present. The patient also received antivenom.

At the end of the intervention, the INVOS (In Vivo Optical Spectroscopy) value of the affected limb was 88 (rSO2); preoperatively, it was undetectable. Daily wound care treatment and vacuum-assisted closure (VAC) dressing are continued. Five days postoperatively, the VAC dressing is removed, and secondary suture of the fasciotomy incisions is performed. The patient has progressed favourably postoperatively and was eligible for discharge, with a recommendation for physiotherapy.

#### 2.2.2. Patient 2—Viper Bite Wound on the Left Hand with Important Oedema and Impaired Range of Motion of the Fingers

**Figure 5:**
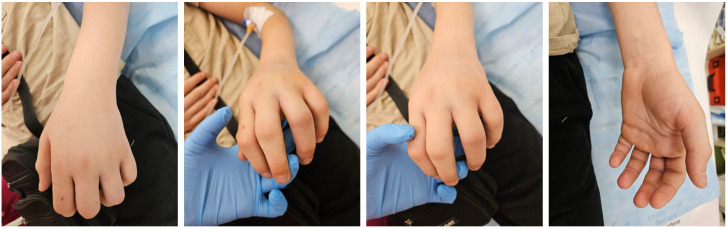
Aspects of the upper limb observed during hospitalisation and treatment: perilesional oedema is observed, without skin colour changes, with preservation of joint contours, normal nail coloration and minimal wound, without secretions.

A 12-year-old patient was admitted to the emergency department with a partial viper bite wound on the 3rd–4th interdigital space on the left hand (Figure 5). Local wound care with antiseptic solutions and dressing was performed.

Local evolution was clinically monitored, and tetanus vaccination was administered. Antibiotics, analgesics, antiallergics, and anticoagulants were administered. The patient’s mother requests discharge, and the hospitalisation period is 3 days. The recommendations were to avoid physical effort and local trauma, keep the hand elevated, and dress the wound with local Microdacyn60^®^ Wound Care until the oedema disappears.

Table 2 presents the results of the investigation for both patients, regarding haematological parameters (complete blood count), coagulation profiles and serum biochemistry analyses.

## 3. Discussion

In Romania, only 3 of the 15 viper species found in Europe are present, and the viper is the only venomous snake. Also, in Romania, snakebites are common in the summer months, during our study period, with peaks in June (5 cases), July (7 cases), and August (5 cases), similar to what has been reported in other studies [5,13].

After a viper bite, highly severe toxic effects include: muscle necrosis; local infections; permanent neurological damage; skin necrosis and defects; and renal failure due to myoglobinuria (rarely as a direct result of SC) [14]. Local effects, including oedema and venom-induced allergic reactions, are most commonly reported after a snakebite, but cases of haematotoxic manifestations are also described [15]. A very rare case described is that of Ludwig’s Angina after a viper bite in a 20-year-old woman with a potentially lethal cellulitis in the oral cavity, with a major risk of airway blockage and death [16].

The viperine syndrome generates a toxidrome that combines coagulation disorders and local toxicity, with sometimes extensive oedema, significant pain, blisters and skin necrosis. Antivenom is administered based on clinical symptoms and the severity classification of viper bites, as defined by Audebert-Moels and modified by Marano et al. [17]. In our study, it was administered to four patients; in cases of severe systemic effects, persistent hypotension, ECG changes, acute toxic pulmonary oedema, coagulopathy with spontaneous bleeding, rhabdomyolysis syndrome, severe metabolic acidosis, etc., 1 ampoule of 500 UA/mL monovalent antivenom produced by the Biomed company, Poland, with specific equine immunoglobulins was administered.

Compartment syndrome in poisoning has a major impact on patients, whether paediatric or adult [18,19]. The mechanism of local toxicity is explained by the fact that viper venom metalloproteinases degrade collagen and basement membrane, leading to plasma and haemorrhagic extravasation; kininogenic toxins increase vascular permeability and cytotoxins induce local cellular apoptosis [6,10,20]. Therefore, the development of compartment syndrome is a consequence of massive oedema, leading to increased intracompartmental pressure, ischaemia, and, at the same time, necrosis [19].

Compartment syndrome after a viper bite is a rare phenomenon, and its treatment remains controversial [21]. Surgical decompression in cases of compartment syndrome after a viper bite is not a common treatment according to the literature. Some authors believe that rapid access to medical care and administration of antivenom may be effective in treating compartment syndrome without the need for fasciotomy [4].

Management of severe oedema of the limbs can be successful with conservative treatment; many cases respond to adequate or repeated antivenom doses, moderate elevation of the limb (not above heart level in severe oedema), adequate analgesia, and close monitoring of compartment pressure and neurovascular status.

Fasciotomy is warranted in selected cases, with compartment pressure > 40 mmHg and progressive clinical deterioration: clear signs of irreversible ischaemia [22]. Unjustified early intervention may worsen local lesions and increase the risk of infection and haemorrhagic complications. However, surgical intervention for compartment syndrome following a viper bite has been reported in developed countries, including France and Italy, in both the upper and lower limbs when indicated [4,23]. In Romania, one case of a 32-year-old woman was described with upper limb compartment syndrome that required fasciotomy [22].

In the case of our first patient described, due to the toxic effects of snake venom, the clinical symptoms were highly suggestive of compartment syndrome evolution; tissue perfusion was reduced and the immediate fasciotomy was necessary. Spontaneous pain on pressure, active and passive extension of the fingers, and increased intracompartmental tension in the lower limb at three successive assessments at 1 h intervals met the criteria for fasciotomy. The lower limb also shows severe skin colour changes (blue and dark blue) in some areas. High CPK levels were also considered; their subsequent decrease demonstrated the effectiveness of fasciotomy.

Antivenom serum was also administered. No immediate or delayed adverse reactions were reported after administration of the antivenom, except for a slight increase in IgE levels without clinical expression. Similarly, it happened in the case of a 6-year-old boy reported in southern Austria, the viper bite being on the forearm, and conservative therapies were not effective. This patient also received antivenom serum [24]. No adverse reactions were observed after antivenom serum administration in a 5-year-old girl reported in Romania, also after a common European adder (*Vipera berus*) bite [3].

Antivenom administration is the therapy of choice, and fasciotomy is indicated selectively only when the situation requires it, based on rigorous clinical and paraclinical evaluation. Fasciotomy in cases of compartment syndrome after a viper bite remains a safe indication when the risks of losing the limb are high, even if there are numerous controversies in the literature regarding this procedure. The indication for fasciotomy remains controversial, as toxic oedema can mimic compartment syndrome without real ischaemia.

The prognosis can be favourable if treatment is initiated as soon as possible, with antivenom administration playing a special role. Antivenom serum is the only specific treatment [25]. The antivenom is effective in both adults and children and is administered at different dilutions [25,26]. It should be noted, however, that serious adverse reactions may also occur when administering antivenom, such as Kounis Syndrome [12]. The administration of antivenom helps both minimise the need for surgery and support postoperative recovery [27].

Fasciotomy was necessary in patient 1; it was performed with an approach from the internal intermuscular septum with continuation to the superficial and deep muscular layer (leg); at the level of the thigh: internal aspect of the knee for decompression of venous return [23].

Biologically, in patient 1, anaemia was evident during hospitalisation (HGB ranging from 6.3 g/dL to 10.1 g/dL), decreased activated partial thromboplastin time (20.8 s), increased CPK (from 589 to 729 U/L), and increased d-dimers (2653 ng/mL), aspects mentioned in other cases also [6,28].

In the first patient, major systemic inflammation, high activated coagulation, and acute anaemia were noted. C-reactive protein (CRP) is elevated at 34.38 mg/L (NV < 5), suggesting severe acute inflammation due to the toxicity of the viper venom, with oedema, severe pain, and local necrosis. The normal erythrocyte sedimentation rate (ESR 4 mm/h) does not exclude acute inflammation. Important to observe is the massive activation of coagulation and fibrinolysis, correlated with severe inflammation, tissue ischaemia and with clinical manifestation of compartment syndrome. Initially, D-dimers have elevated values (2653 ng/mL), later returning to normal 11 ng/mL, due to the toxic activity of metalloproteinases and serine proteases from the venom; APTT 20.8 s (NV 23–34) suggests a procoagulant status. Normalisation of D-dimers confirms the effectiveness of the administered treatment.

Also very relevant is the severe muscle damage with progressive rhabdomyolysis. CPK with high values of 589/729/354 U/L (VN < 225) and increased LDH 478 U/L (NV < 300) can be interpreted in the context of local ischaemia, due to increased compartmental pressure, muscle ischaemia, justifying the indication for fasciotomy.

Analysis of the dynamic evolution of the haemoleukogram. Leukocytes are initially 12.9 (neutrophilia), then normalisation to 7–9 occurs in an acute inflammatory context. Hb with low values of 6.3 g/dL and Ht with values of 18–28% indicate a severe acute anaemia, probably due to tissue loss, haemodilution or a major inflammatory context, frequently described in severe bites in children. Platelets with initially elevated values of 611,000 are specific for reactive thrombocytosis in the context of severe inflammation as a toxic response to venom. A slight increase in glycaemia may be explained by metabolic stress (123 mg/dL).

Following surgery, negative-pressure dressings were applied. The role of using a vac is to reduce the spread of venom into tissues, facilitate the return of interstitial fluid to the absorbing sponge rather than to the lymphatics, and limit its spread to the circulatory system. Also, negative pressure helps eliminate inflammatory cytokines released in the tissue. The role of VAC is to modulate cytokines, reducing tissue oedema and increasing vascular flow; references also support its active role in decreasing serum levels of pro-inflammatory cytokines such as IL-6 and TNF-α [29,30].

Negative pressure maintains capillary permeability, limiting the occlusive effect of oedema and the microcirculatory disorders induced by venom-induced peripheral microthrombosis. In this way, negative pressure favours both the elimination of venom that has not yet been absorbed into the tissues and the interruption of toxic phenomena induced by venom. The expulsion of oedematous fluid and the physical absorption of snake venom through VAC are also described by Zeng F, which reduces systemic reactions after venom absorption and plays an important role in improving swelling and venom drainage [30]. To allow safe healing of the tissues affected by venom, the cow was maintained for at least 5 days before attempts to close the wounds were initiated, and subsequent wound closures were performed every 2 to 3 days.

Patient 1 was discharged on day 6 without clinical signs or symptoms. Laboratory test results were within normal ranges (see Table 1). Summary urine and sediment were analysed, and the parameters were normal in both patients: protein (5.5 mg/dL), bilirubin (negative), urobilinogen (normal), ketone bodies (negative), ascorbic acid, glucose, protein (negative), etc.

For patient 2, laboratory investigations reveal the absence of systemic inflammation: CRP: 0.34–0.43 mg/L (N) and ESR: 3 mm/h (N). The venom’s toxic effects are locally limited, with a low risk of severe systemic toxicity. There is no venom-induced coagulopathy: D-dimers < 100 ng/mL (N), normal APTT, INR 1.23–1.24 (upper limit, clinically insignificant), and normal Fibrinogen. Muscle involvement is also absent, CPK: 159 → 85 U/L (N), with normal LDH. Haemogram shows normal WBC, relative neutrophilia, and discrete lymphopenia in response to stress and pain in the acute phase.

Therefore, patient 2 presents with a viper bite of minimal to moderate severity, characterised by mild local manifestations, no systemic inflammation and coagulopathy, and a favourable evolution under supervision without surgical intervention. Symptomatic treatment was effective for patient 2, and after three days of hospitalisation, he was discharged with a good prognosis.

Recognition of the viper is possible only if the owners can describe it or bring a dead specimen to the hospital; in the cases presented, the viper was identified based on descriptions provided by patients, parents, and local authorities, as well as on the presence of vipers in well-known areas. The Suceava area, for example, is known for its high viper population. In addition to recognising the viper, it is also important to describe the viper bite, which often appears as a 2-point wound at a distance of 5–12 mm, with the skin becoming purple and blisters forming. Also, the bite can present as a single punctate sting or one/two parallel abrasions from the two retractable teeth [31]. The volume of venom inoculated is important in the development of complications and varies with the depth of the bite, the snake’s size, and the amount of venom in the snake’s glands [31]. Also, the composition of the venom is important because it contains numerous enzymes, including proteases, hyaluronidases, and phospholipases, with variable specificities [10,32]. Viper venom is haemotoxic, cytotoxic [33] and sometimes neurotoxic [3]. Paolino et al. describe the implications of phospholipases A2 (vipoxin, ammodytoxins) in neurotoxicity and myotoxicity, of metalloproteinases in haemorrhage and vascular destruction, of serine proteases (thrombin-like enzymes) in coagulopathy, and of activators of FX/fibrinolysis (ammodytase) in hypofibrinogenemia and DIC [20,34]. They can even cause necrosis by destroying tissues in the vicinity of the viper bite. The actions of viper venom enzymes can explain coagulopathies at different stages of haemostasis, which frequently replace physiological enzymes [14,34]. Close to Romania, in Bulgaria, a case of severe coagulopathy following a *Vipera ammodytes ammodytes* snakebite, a species that is toxinologically significant for humans, was reported in a 56-year-old man with severe thrombocytopenia [4]. The effects of snake venom on the human cytokine network were studied [33]. Future research directions may also include determining venom composition using UHPLC-MS/MS if patients bring the dead exemplar to the hospital.

There remains a need to improve awareness and highlight prevention strategies, particularly among children, such as intensifying education on snakebites in rural schools and encouraging the use of appropriate footwear, as noted in other scientific papers [15].

## 4. Conclusions

In Romania, viper bites represent important medical emergencies that can evolve clinically and can be life-threatening. This study analyses 24 cases of viper bites in paediatric patients encountered over 10 years (2016–2025) at Saint Mary Emergency Hospital, Iasi, Romania, highlighting the patterns of clinical toxicity and surgical complications. The mean age of the patients was 11 years (SD = 5). The mean length of hospital stay was 4 days (SD = 2). There was a statistically significant correlation between a shorter length of hospital stay following antivenom administration. The majority of cases involved bites to the lower limbs and predominantly local symptoms; however, two patients required plastic surgery monitoring, and one developed compartment syndrome, which required fasciotomy and VAC therapy. The study emphasises that compartment syndrome following paediatric viper bites requires prompt, multidisciplinary management for limb salvage. In paediatric patients, viper bites can be associated with severe local effects and, although rare, may lead to compartment syndrome that requires a rigorous surgical approach to prevent loss of function of the affected limb.

Based on the data presented in this study, we conclude that viper bites remain a public health concern in Northeastern Romania. Correct and multidisciplinary management can considerably improve patients’ functional prognosis. This paper aims to raise awareness of the toxicological complexity of viper bites and to reduce the number of such cases. It would also be beneficial to develop guidelines on post-viper-bite behaviour for educational and preventive purposes.

## 5. Materials and Methods

We conducted a 10-year retrospective study (2016–2025) of paediatric patients (<18 years) with viper bites evaluated at the emergency reception unit, some of whom were managed in collaboration with the Plastic Surgery Clinic at the Children’s Emergency Clinical Hospital, Iasi, Romania. The current study adhered to the Declaration of Helsinki and was approved by the Ethics Committee of Saint Mary Emergency Hospital in Iasi, Romania (Approval number 35804/15 October 2025). Patient data were anonymised, and participants’ parents signed a consent form upon admission to the hospital for the anonymous use of their data for research purposes.

After the Ethics Committee approved the protocol, the patients’ medical records were analysed. The data from the statistics department were retrieved and evaluated. Also, the information present in the emergency department—the date of presentation, demographic data, the symptoms at the time of presentation of the patients and whether or not the administration of anti-viper or anti-tetanus serum was necessary—was analysed. Furthermore, the cases requiring plastic surgical management were analysed and described in detail, with all the data available (pictures, investigations, etc.). Data were extracted from emergency reception unit charts, observation sheets and surgical registers. Demographic data, bite location, clinical severity, paraclinical investigations and treatment were analysed.

Patient characteristics and hospitalisation trends were analysed using IBM SPSS Statistics (version 26.0), and the hospitalisation flow was visualised as an alluvial diagram in the Flourish programme. Descriptive statistics were used to compute the mean, median, minimum, and maximum values for two parameters: age and length of hospital stay.

The primary outcome was represented by surgical complications (compartment syndrome, tissue necrosis, fasciotomy, etc.). Secondary outcomes included length of hospitalisation, clinical symptomatology at presentation, demographic data, adverse reactions to antivenom, and other outcomes. Patients < 18 years with a clinical diagnosis of viper bite were included; other cases of animal/insect bites were excluded from the analysis.

## Figures and Tables

**Table 1:** Sociodemographic and medical characteristics at admission.

Parameter		N	%
Area of residence	Rural	19	79.2
	Urban	5	20.8
Sex	F	5	20.8
	M	19	79.2
Complications	No	15	62.5
	Yes	9	37.5
Surgical intervention	No	23	95.8
	Yes	1	4.2
Tetanus vaccine	No	5	20.8
	Yes	19	79.2
Antivenom	No	20	83.3
	Yes	4	16.7
Limb	Lower extremity	11	73.3
	Upper extremity	4	26.7

**Table 2:** Investigation results for the patients (haematology/blood count, coagulation and serum biochemistry).

Investigations	Reference Range	Patient 1			Patient 2	
		Day 1	Day 3	Day 5	Day 1	Day 2
Haematology—Blood count						
WBC (× 10^3^/µL)	4.5–13.5	12.94	9.5	7.46	6.97	5.34
NEUT# (× 10^3^/µL)	1.8–8	9.87	5.69	4.28	5.59	2.94
NEUT% (%)	40–75	76.2	59.9	57.4	82	55.1
LYMPH# (× 10^3^/µL)	1.5–6.5	2.34	2.71	2.27	1.15	1.82
LYMPH% (%)	20–55	18.1	28.5	30.4	15.6	34.1
MONO# (× 10^3^/µL)	0–1	0.71	0.96	0.52	0.21	0.52
MONO% (%)	0–15	5.5	10.1	7	3	9.7
EO# (× 10^3^/µL)	0–0.6	0	0.1	0.36	0	0.04
EO% (%)	0–7	0	1.1	4.8	0	0.7
BASO# (× 10^3^/µL)	0–0.2	0.02	0.04	0.03	0.02	0.02
BASO% (%)	0–2	0.2	0.4	0.4	0.3	0.4
IG# (× 100^3^/µL)	0–0.10	0.05	0.06	0.05	0.01	0.01
IG% (%)	0–1	0.4	0.6	0.7	0.1	0.2
RBC (× 100^3^/µL)	4.1–5.2	5.07	2.36	3.65	4.85	4.28
HGB (g/dL)	11–14	13.5	6.3	10.1	13.6	12.2
HCT (%)	35–45	38.3	18.2	28.7	40.2	35.1
MCV (fL)	77–94	75.5	77.1	78.6	82.9	82
MCH (pg)	26–32	26.6	26.7	27.7	28	28.5
MCHC (g/dL)	32–37	35.2	34.6	35.2	33.8	34.8
RDW-CV (%)	10–15	12.9	13.1	12.5	12.7	12.9
RDW-SD (fL)	33–49	34.5	35.8	35.6	38.5	38.7
PLT (× 10^3^/µL)	150–400	611	243	240	233	163
MPV (fL)	8.5–12	9	9	9.4	10	10.3
PCT (fL)	0.12–0.44	0.55	0.22	0.23	0.23	0.17
P-LCR (%)	14.3–44	16.9	15.9	19.2	26.6	26.8
PDW (fL)	10–18	9.9	8.7	9.5	11.7	10.9
Coagulation						
FIB (sec)		18.4			20.2	18.8
FIB (mg/dL)	180–400	330.9			263.7	287.5
APTT (sec)	23–34	20.8			26.4	34.2
PT (sec)	13–17	21.4			21.4	20.9
INR	0.8–1.2	1.22			1.24	1.23
PT (%)	70-100	80.6			79.8	80.2
D-dimers (ng/mL)	0–250	2653			<100	
Serum biochemistry						
ALT/TGP (U/L)	5–38	5			12	
AST/TGO (U/L)	5–35	30			25	
Creatinine (mg/dL)	0.1–0.7	0.39			0.59	0.59
C-reactive protein (CRP)		34.38			0.34	
Creatine phosphokinase (CPK) (U/L)	24–225	589	729	354	159	85

WBC—white blood cell count; NEUT#/NEUT%—absolute neutrophil count and percentage; LYMPH#/LYMPH%—absolute lymphocyte count and percentage; MONO#/MONO%—absolute monocyte count and percentage; EO#/EO%—absolute eosinophil count and percentage; BASO#/BASO%—absolute basophil count and percentage; IG#/IG%—absolute immature granulocyte count and percentage; RBC—red blood cell count; HGB—haemoglobin concentration; HCT—haematocrit; MCV—mean corpuscular volume; MCH—mean corpuscular haemoglobin; MCHC—mean corpuscular haemoglobin concentration; RDW-CV—red cell distribution width (coefficient of variation); RDW-SD—red cell distribution width (standard deviation); PLT—platelet count; MPV—mean platelet volume; PCT—plateletcrit; P-LCR—platelet large cell ratio; PDW—platelet distribution width.

## Data Availability

The original contributions presented in this study are included in the article. Further inquiries can be directed to the corresponding author(s).

## Abbreviations

The following abbreviations are used in this manuscript:

N	Number
NV	Normal values
